# The importance of physical activity in management of type 2 diabetes and COVID-19

**DOI:** 10.1177/20420188211054686

**Published:** 2021-10-26

**Authors:** Samuel Seidu, Kamlesh Khunti, Tom Yates, Abdullah Almaqhawi, M.J. Davies, Jack Sargeant

**Affiliations:** Diabetes Research Centre, University of Leicester, Leicester LE5 4PW, UK; Diabetes Research Centre, University of Leicester, Leicester, UK; Diabetes Research Centre, University of Leicester, Leicester, UK; Department of Family and Community Medicine, College of Medicine, King Faisal University, Dammam, Saudi Arabia; Diabetes Research Centre, University of Leicester, Leicester, UK; Diabetes Research Centre, University of Leicester, Leicester, UK

**Keywords:** COVID-19, diabetes, exercise, management

## Abstract

Over time, various guidelines have emphasised the importance of physical activity and exercise training in the management of type 2 diabetes, chronic diseases, including cardiovascular disease and musculoskeletal disorders. The aim of this review is to evaluate the effectiveness of physical activity in people with type 2 diabetes and COVID-19. Most research to date indicates that people with type 2 diabetes who engage in both aerobic and resistance exercise see the greatest improvements in insulin sensitivity. Physical activity is now also known to be effective at reducing hospitalisation rates of respiratory viral diseases, such as COVID-19, due to the beneficial impacts of exercise on the immune system. Preliminary result indicates that home-based exercise may be an essential component in future physical activity recommendations given the current COVID-19 pandemic and the need for social distancing. This home-based physical exercise can be easily regulated and monitored using step counters and activity trackers, enabling individuals to manage health issues that benefit from physical exercise.

## Introduction

The COVID-19 pandemic has occurred in conjunction with a worsening type 2 diabetes pandemic, which the world has been struggling to control for many decades. Diabetes mellitus is a clinical condition characterised by abnormal glucose metabolism and hyperglycaemia due to absolute or relative insulin deficiency, insulin resistance or both.^1^ Diabetes is becoming more common in the United Kingdom, with 3.9 million people currently diagnosed with diabetes and 90% of those with type 2 diabetes.^2,3^ This figure is expected to increase to over 5.3 million by 2025.^2,3^ As a result of the increased occurrence of diabetes, it is now the fourth leading cause of noncommunicable diseases (NCDs) worldwide.^4^ In 2018–2019, 55 million pharmacological interventions were administered, up from 33 million a decade earlier.^5^ The National Health Service (NHS) invests at least £10 billion a year (approximately £27 million per day) on diabetes, which equates to 10% of its overall expenditure, with 80% spent on complications.^6^ Type 2 diabetes is caused by glucose dysregulation, lipid metabolism and metabolism due to decreased insulin production, insulin resistance or both and is generally more common in people who do not do enough physical activity, are overweight or both.^7^

The World Health Organization (WHO) announced a public health emergency on 30 January 2020, in response to the spread of the novel coronavirus (2019-nCoV).^8^ Severe acute respiratory syndrome coronavirus 2 (SARS-CoV-2) is a novel virus known to cause COVID-19^9^ and usually begins with an asymptomatic incubation period (5–6 days, maximum range: 1–14).^10^ COVID-19 is a mild illness in about 80% of cases, most often accompanied by fever and cough, but other symptoms may include sore throat, nausea, shortness of breath, headache, myalgia, loss of smell and taste, chills, vomiting and diarrhoea.^11^ According to recent data (3 September 2021), more than 6,862,904 people in England have been infected with COVID-19.^12^

In hospitalised patients with COVID-19, diabetes has been described as a significant risk factor for mortality and high rates of progression to acute respiratory distress syndrome.^13^ COVID-19 patients with diabetes need more medical interventions, have higher mortality rates [7.8% *versus* 2.7%, adjusted hazard ratio (HR): 1.49] and have greater frequency of multiple organ damage than those without diabetes.^14^ Hyperglycaemia raises the expression of tumour necrosis factor-alpha (TNF-α) and interleukin-6 (IL-6), which aggravates inflammation.^15^ In studies involving other viruses, a higher proclivity to infection is observed due to the presence of inflammatory processes.^16^

Healthy People 2020 objectives and UK National Institute for Health and Care Excellence (NICE) recommendations emphasise the importance of increasing physical activity as part of the treatment of many chronic diseases, including cardiovascular disease, diabetes and musculoskeletal disorders.^17,18^ Many studies have shown that physical activity has significant health and disease benefits, including lowering mortality rates.^19^ In people living with type 2 diabetes, physical activity has been linked to reduced systolic pressure, lowered risk of diabetes-related complications and diabetes-related death and myocardial infarction.^20^ Moreover, evidence suggests physical activity reduces the risk factors related to the development of type 2 diabetes.^21^ Notably, a systematic review and meta-analysis investigated the impact of routine exercise training on dynamic insulin sensitivity measures in adults with type 2 diabetes and found that insulin sensitivity was significantly higher in the exercise group than in the control group and this effect lasted for up approximately 72 h after the last training event.^22^

Physical inactivity is correlated with a higher relative risk of COVID-19 hospitalisation, even after controlling for age, sex, obesity, smoking and alcohol intake [relative risk: 1.32 (95% confidence interval [CI]: 1.10–1.58)], suggesting that physical inactivity may also increase COVID-19 hospitalisations.^23^ The mechanisms behind this association are likely multifactorial, although they might include beneficial effects of physical activity on the immune system.^24^ Physical inactivity has a massive effect on global health, causing 7.2% of type 2 diabetes cases and 9.4% of all-cause mortality worldwide.^24^ The prevalence of physical inactivity among people at risk of type 2 diabetes is estimated to be 43.2%.^24^ Globally, a recent survey during the COVID-19 pandemic suggested the prevalence of physical inactivity was 57.3% and 57.7% among the general population and individuals at risk for type 2 diabetes, respectively.^25^

Given these results, increasing physical activity may likely have a positive impact on public health and reduce financial resources wastage during the COVID-19 pandemic. This review highlights the role of physical activity in people with type 2 diabetes and COVID-19. The review begins with a summary of the literature on the health benefits of physical activity, followed by a review of studies reporting the links between physical activity and diabetes and the relationship between physical activity and COVID-19.

## Physical activity, types and known benefits

**Figure 1.**
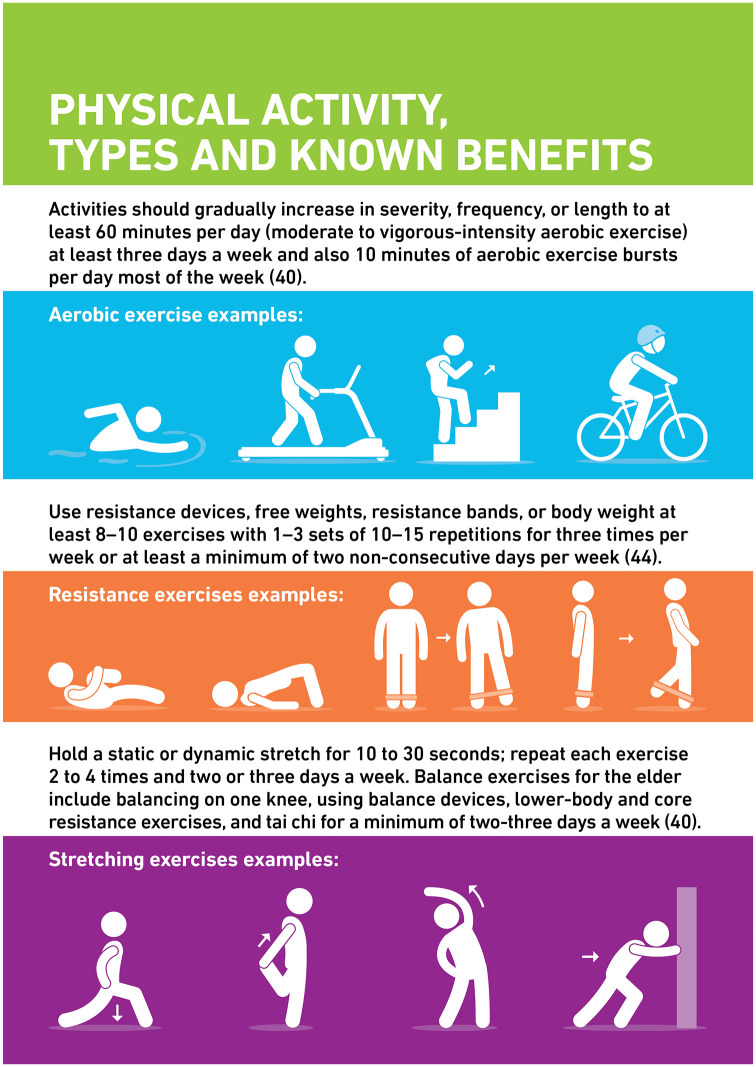
Physical activity types and known benefits.^27,29^

Physical activity can take several forms. Aerobic exercises are activities that use larger muscles that rely primarily on energy provided by aerobic metabolism. These exercises are suggested to enhance the quality of life in individuals with type 2 diabetes (Figure 1). Aerobic training, which may include activities such as running, jogging, cycling and swimming, can vary from moderate to vigorous in intensity that typically last 20 min.^26,27^ High-intensity interval training consists of repeated periods of intense exercise separated by intervals of rest.^28^ Resistance training consists of exercises aimed at increasing muscle strength and stamina using bodyweight, devices or resistance bands.^26^ Flexibility and balance are essential for preserving joint range of motion and might to be useful for people with diabetes. Yoga, upper and lower body stretches are examples of stretching exercises that will help an individual become more flexible.^26^

The benefits of physical activity for acute and chronic health conditions are well studied, including its positive influence on blood glucose levels in people with type 2 diabetes. It was suggested in two different studies that most of the improvements in insulin action were attributed to the acute effects of a recent bout of exercise, with most people reporting a drop in their blood glucose levels during low- to moderate-intensity exercise and for 2–72 h afterwards.^30,31^ Acute enhancements in insulin sensitivity have also been observed in women with type 2 diabetes who participate in either low-intensity or high-intensity walking for comparable energy expenditure.^32^

Furthermore, a review study reported that exercise interventions alone have been effective in avoiding increases in blood glucose, body weight, lipids, blood pressure, cardiovascular disease and mortality and raise the overall quality of life in type 2 diabetes patients.^33^ A randomised control trial recently examined the impact of 8 weeks of high-intensity training focussed on functional and multijoint motions on glucose control and body composition in overweight or obese adults. The adult sedentary type 2 diabetes group showed a substantial increase in β-cell activity after 10–20 min of training 3 times a week, a decrease in fat storage, maintenance of body weight, increased cardiovascular health, lipid metabolism and low-density lipoprotein cholesterol as well as enhanced insulin sensitivity.^34^ Another study followed 13 overweight and obese adults with type 2 diabetes for 6 weeks who participated in functional high-intensity training that included aerobic and resistance training varying from 8 to 20 min per session 3 days a week. The results showed a substantially reduced fat mass and adiponectin, diastolic blood pressure, blood lipids and metabolic syndrome and increased basal fat oxidation and high molecular weight adiponectin. The conclusion drawn from the study was that functional high-intensity training enhanced insulin sensitivity.^35^

A 16-week multicentre randomised controlled trial assessed exercise in the prevention of metabolic syndrome and found that low-volume, high-intensity interval training (51 min/week) was as effective as high-volume, high-intensity interval training (114 min/week) and moderate-intensity continuous training (150 min/week) in minimising metabolic syndrome severity.^36^ Research also suggests that replacing sedentary behaviour with light-intensity physical activity could be effective for reducing diabetes risk markers.^37^ Even one session of aerobic exercise per week, 50 min of treadmill walking at 70% of maximum oxygen consumption will boost whole-body insulin sensitivity in people with type 2 diabetes.^38^ Therefore, it is possible that resistance exercise training also improves blood sugar regulation and insulin action in people with type 2 diabetes.^39,40^ In a randomised control trial, older men with newly diagnosed type 2 diabetes who did twice-weekly progressive resistance training for 16 weeks showed a 46.3% rise in insulin action, 7.1% drop in fasting blood glucose levels and a substantial loss of abdominal fat.^41^ Another randomised control trial aimed to determine how low-volume, high-intensity interval training affected cardiometabolic risk and exercise ability in women with type 2 diabetes.^28^ The key finding was that the low-volume, high-intensity interval training programme successfully lowered fasting glucose and HbA1c levels over 16 weeks, despite the daily dose of glucose-lowering therapies being reduced.^28^ The low-volume, high-intensity interval training programme also improved lipid profile, blood pressure, endurance performance and body composition.^28^

Several studies have highlighted that aerobic exercise enhances mitochondrial density, insulin sensitivity, oxidative enzymes, blood vessel compliance and reactivity, lung function, immune function and cardiac performance among people with type 2 diabetes.^42^ In adults with type 2 diabetes, high-intensity interval training facilitates rapid improvements in skeletal muscle oxidative ability, insulin sensitivity and glycaemic control.^28,43^ Interestingly, short bouts of near-maximal intensity aerobic exercise (20 min) result in post-exercise insulin action improvements lasting up to 24 h.^27^ Therefore, aerobic exercise may improve functional capacity, fitness and health-related quality of life in people with type 2 diabetes.^44^

Both aerobic and resistance exercise have been shown to enhance skeletal muscle, adipose tissue and liver health and control insulin sensitivity, which are related to weight loss. Aerobic exercise, however, showed a slightly greater significant reduction in HbA1c (difference of 0.18%) compared with resistance exercise.^27^

One systematic review assessed the impact of resistance exercise of varying intensities on HbA1c, insulin and blood glucose levels in people with type 2 diabetes.^29^ After resistance exercise, there was a correlation between intensity and resistance training resulted in reduced HbA1c (*p* = 0.006) and insulin (*p* = 0.015) levels in a meta-regression study. Subgroup analysis indicated that high-intensity exercise was associated with lower HbA1c than low- to moderate-intensity exercise.^29^ In addition, the differences in HbA1c and insulin levels between the subgroups were statistically significant, indicating that participation in high-intensity resistance exercise has significant benefits.^29^ Standing on one foot, tai chi and heel-to-toe walking are some exercises that can help people improve their balance to avoid falling.^26^ Stretching improves range of motion and flexibility around joints; it, however, does not affect glycaemic control.^45^ Likewise, balance training helps reduce the risk of falling by improving balance and gait, even when peripheral neuropathy is present.^46^ Tai chi training in diabetes and neuropathic patients will enhance glycaemic regulation, balance, neuropathic symptoms and some qualitative aspects of life in adults with diabetes and neuropathy.^47^ Similarly, increased unstructured physical activity (e.g. errands, housework, dog walking or gardening) improves everyday energy expenditure and weight control.^48,49^ Walking activity effectively reduces postprandial hyperglycaemia and improves glycaemic control in people with prediabetes and type 1 and type 2 diabetes, particularly after meals.^50–52^

## SARS-CoV-2 virus and mechanisms

SARS-CoV-2 has a single-stranded positive RNA and a transmembrane spike glycoprotein (S protein).^53^ Both S protein binding to a cellular receptor and S protein priming by a cellular protease are needed for cell entry.^54^ SARS-CoV-2 has a large number of glycosylated S proteins on its surface that bind to the host cell receptor angiotensin-converting enzyme 2 (ACE2), mediating viral cell entry.^55^ The process of clearing the virus and reducing lung damage by directing monocytes, macrophages, virus-specific T cells and natural killer (NK) cells to the infection site is attracted by the initial inflammation.^56,57^ Similarly, neutralising antibodies can prevent viral infection,^51^ and alveolar macrophages recognise neutralised viruses in dying cells and clear them through phagocytosis, which has the same effect on stimulating the immune response.^58,59^

Conversely, compromised immune response contributes to infection spread, especially to the lower respiratory tract, resulting in severe COVID-19 disease outcomes.^9,60^ Increased disease progression caused by an excessive and dysregulated inflammatory response can trigger tissue damage at the point of virus entry. Several factors have been linked to muscle atrophy and sarcopenia, especially during the COVID-19 pandemic, including an increase in interferon-gamma (IFN-γ), interleukin-1β (IL-1β), IL-6, interleukin-17 (IL-17) and tumour necrosis factor (TNF), corticosteroid therapy and mechanical ventilation and physical inactivity as a result of public health guidelines for quarantine.^61–63^ Approximately 15% of COVID-19 cases become moderate, profound and progress to severe pneumonia, primarily due to a compromised immune response, and approximately 5% experience acute respiratory distress syndrome, septic shock or multiple organ failure.^64^

## COVID-19 and diabetes

Data from several studies suggest that diabetes is linked to an increased risk of various infections.^65^ Specifically, an increased risk of infection was identified during previous outbreaks of severe acute respiratory syndrome, Middle East respiratory syndrome and H1N1 influenza virus.^66,67^ Recently, diabetes has been noted to be more prevalent in patients with severe COVID-19. A recent meta-analysis of COVID-19, which included eight studies, showed people with diabetes had a higher chance of intensive care unit (ICU) admission.^68^ It has now been suggested in a retrospective study of COVID-19 patients that people with diabetes had a higher incidence of hypertension (56.9%), cardiovascular disease (20.9%) and cerebrovascular disease (7.8%) than those without diabetes (28.8%, 11.1% and 1.3%, respectively).^69^ Furthermore, nonsurvivors of COVID-19 with diabetes have a higher occurrence of comorbidities than survivors (hypertension: 83.9% *versus* 50.0%; cardiovascular disease: 45.2% *versus* 14.8%; cerebrovascular disease: 16.1% *versus* 5.7%; chronic pulmonary disease: 12.9% *versus* 3.3% and chronic kidney disease: 6.5% *versus* 3.3%).^69^

## COVID-19 and physical activity

A strong positive relationship between physical activity level and immunity defences has been shown in several studies. Codo *et al.*^70^ demonstrated several aspects of the immune response being positively associated with physical exercise. Exercise training is associated with more effective immune defence and better glycaemic control, both of which can play a role in SARS-CoV-induced immune cell activation.^70^ Physical activity has been shown in some studies to boosts immunity against infectious pathogens, such as viruses, as physical activity and exercise induce significant movement of leukocytes in blood and tissues.^71^ Acute exercise is also thought to be an essential stimulus for increasing CD34+ haematopoietic stem cells and mobilising cell-mediated immunity, as shown by a two- to fivefold increase in the blood circulating leukocytes.^72^ Several authors have considered the effects of exercise on increasing NK cell mobilisation, boosting neutrophil chemotaxis and phagocytosis and modulating inflammatory/alternative activated macrophages in adipose tissue.^73,74^ Physical activity has also been linked to improved immune response by reducing fat tissue, which facilitates the enhancement of low-grade chronic inflammation.^75^

Regular exercise for at least 6 months has been shown to prevent age-related immune dysfunction/immunosenescence, chronic low-grade inflammation and increase flu vaccination efficacy in elderly populations without causing harm.^76^ Because the elderly are more prone to infection, which might lead to significant health risk, the benefits of exercise on immune function might be particularly important among those with COVID-19. Several epidemiological studies have shown that daily physical exercise correlates with lower influenza and pneumonia mortality and incidence rates.^77^ This actively demonstrates that physical activity is a successful strategy against hospitalisation rates of respiratory viral diseases, such as COVID-19, and enhances the immune system. Regular physical activity may therefore help to prevent the onset of severe COVID-19 disease by increasing the activation of NK and CD8+ T cells, which would boost the immune response to SARS-CoV-2.^78^ A prospective longitudinal study revealed a substantial association between the balance and variability in the timing of exercise and rest was more closely correlated with the risk of testing positive for SARS-CoV-2 or the rate of severe COVID-19 than ‘normal’ measures of activity, such as moderate to intense physical activities.^79^ It has been suggested that people who met physical activity requirements on a regular basis, or even those who did some physical activity, were less likely to be hospitalised, admitted to the ICU and die than those who were physically inactive during the 2 years prior to the pandemic.^80^ Physical inactivity was also the greatest risk factor for severe COVID-19 outcomes, after advanced age and a history of organ transplant.^80^ More recent attention has focused on the number of steps per day and other exercise measures, as practicing regular physical activity encourages cardiovascular health and improved exercise stress testing outcomes.^81^

## Importance of physical activity to diabetes management in a COVID era

### Summary of the potential importance of exercise those with diabetes the COVID era

As shown above, diabetes seems to exacerbate the deleterious effects of SARS-CoV-2 infection while physical activity may act in the opposite direction by mitigating or lowering the risk of developing severe infection. Therefore, physical activity is likely to play an important role in managing diabetes within the COVID era. Because the difficulties associated with regulating blood glucose depend on diabetes type, activity type and the prevalence of diabetes-related morbidities, exercise strategies should be personalised to meet each individual’s unique needs. The current circumstances with COVID-19 restrictions pose additional challenges to meeting/maintaining recommended physically active levels.^27^ Notably, diabetes causes muscle strength and functional decline; thus, resistance exercise is needed to improve muscle mass, body composition, fitness, bone mineral density and metabolic constants.^82^

In light of the recommendations for social distancing during the COVID-19 crisis, home-based exercise programmes have emerged as a viable way to monitor, sustain and improve physical activity practice during the pandemic.^83^ Individual activities, flexible scheduling and everyday home routines are all potential advantages of home-based training, which has been shown to be safe and beneficial for people with diabetes.^83^ Dancing, playing with children, cleaning and gardening, as well as participating in an online exercise class, are all ways to remain healthy at home.^84^

An experimental study examined whether small doses of intense exercise before each main meal (referred to as ‘exercise snacks’) resulted in better blood glucose regulation than a single bout of extended, constant, moderate-intensity exercise in people with insulin resistance.^85^ When compared with a single 30-min bout of moderate, continuous exercise before the evening meal, exercise snacking (doing brief bursts of intense exercise immediately before breakfast, lunch and dinner) decreased postprandial and subsequent 24-h glucose concentrations in an insulin-resistant population.^85^ A bout of continuous exercise, on the contrary, did not lower postprandial glucose after dinner or boost glycaemic regulation the next day.^85^

Another randomised clinical trial study investigated and compared the results of supervised group exercise therapy and home-based exercise therapy in Iranian women with type 2 diabetes over 12 weeks.^86^ The supervised group exercise therapy programme resulted in improved quality of life and reduced cardiovascular risk factors and anthropometric parameters than the home-based exercise therapy programme among diabetic women.^86^ Nonetheless, the home-based exercise therapy programme produced a variety of positive results, including for lipid profile, HbA1c and fasting blood glucose.^86^

### COVID-19 pandemic’s effect on physical activity levels in people with diabetes

Physical activity is an essential part of existing UK guidance on diabetes prevention and treatment.^87^ A retrospective study investigated trends of smartphone-tracked behaviour before, after and immediately after a lockdown in the United Kingdom, which concluded that significant reductions in physical activity were observed at all-time points.^88^ People with type 2 diabetes already have poor cardiometabolic health and are less active and more sedentary than those without the disease.^86^ COVID-19 has further negative impacts on physical activity levels, which may have an adverse effect on well-being and health. In an observational analysis in adults with type 2 diabetes, physical activity (measured using objective accelerometery) was lower during COVID-19 restrictions than before the pandemic.^87^ A recent cross-sectional study investigated changes in physical activity levels during self-quarantine in Italy and the effect of exercise on psychological well-being. The study concluded that overall physical activity declined dramatically before and after the COVID-19 pandemic, having a profoundly negative effect on psychological health and well-being.^88^ Similarly, a recent study showed a high percentage of physical inactivity before the COVID-19 lockdown in Spain, which was exacerbated during home confinement.^89^ Advanced age, chronic illness and physical inactivity prior to social isolation were all associated with a higher risk of decreased physical activity levels and increased sitting time during the COVID-19 pandemic.^90^

### General recommendations for maintaining/increasing physical activity levels during COVID

The American College of Sports Medicine (ACSM) recommendations focus on moderate-intensity aerobic activity performance. Specifically, they recommend implementing exercises to retain or improve muscular strength and endurance for at least 2 days per week.^89^ The ACSM suggest adopting moderate-intensity exercises that meet a heart rate reserve or oxygen uptake of 40–59%, perceived exertion rating of 12–13% and 50–69% of one-repetition maximum, no less than 30 min in length and at least 5 days a week is a safe way to encourage well-being in various clinical conditions.^89^ Aside from remaining active during the day with everyday activities, 20 min of intense physical activity at 60–89% heart rate reserve and 70–84% of one-repetition maximum is recommended at least 3 times a week.^89^ To meet the recommended goal, individuals should combine moderate- and vigorous-intensity physical activity corresponding to the demand of at least 500–1000 metabolic equivalent of task (MET) minutes per week, which corresponds to at least 5400–7900 steps/day or approximately 4–6 km.^89^ Individuals are encouraged to train each major muscle group for 2–4 sets of 8–12 repetitions with 2- to 3-min rest periods between sets and 48 h between sessions.^89^ To increase muscular strength, the recommended intensity ranges from 60% to 70% of one-repetition maximum for mild intensity to 80% for vigorous intensity.^89^ Furthermore, the ACSM recommends individuals engage in a series of stability exercises for each significant muscle-tendon category (holding static for 10–30 s) and neuromotor activities for 20–30 min at least 2 days per week to improve balance, strength, coordination and gait.^89^

Exercise and Sport Science Australia suggests that patients with type 2 diabetes mellitus or prediabetes should plan for 210 min of moderate-intensity exercise or 125 min of vigorous-intensity exercise each week.^90^ Resistance training should be used in 2 or 3 sessions a week.^90^ Aerobic and resistance training can also be completed in a single session with 2–4 sets of 8–10 repetitions.^90^ Generally, exercise should be completed at least 3 times a week, with no more than 2 days off in a row.^90^ If required and clinically practicable, the exercise guideline can be met with a combination of moderate- and vigorous-intensity exercise.^90^ This overall amount of exercise can include both aerobic and resistance training. Therefore, the advice is to practice 40 min of moderate intensity and another day for 30 min at a vigorous intensity.^90^

According to the American Diabetes Association, younger individuals with type 1 or 2 diabetes should engage in low- to high-intensity aerobic exercise for 60 min or more a day.^27^ Resistance training should be performed at least 3 times a week to improve muscle strength and bone density.^27^ Adults with type 1 or 2 diabetes are recommended to engage in 150 min or more of moderate- to high-intensity aerobic exercise each week, over at least three sessions per week.^27^ Depending on individual fitness levels, high-intensity or interval training of shorter duration can replace longer moderate-intensity activity at least 75 min per week. Two to three sessions per week of strength training on nonconsecutive days are also recommended.^27^ For older adults with diabetes, flexibility and balance training, such as yoga and tai chi, are recommended 2–3 days a week to improve flexibility, muscle ability and balance.^91^

Promoting the adoption and maintenance of physical activity is also an essential factor to increase physical activity in adults with type 2 diabetes. According to the American Diabetes Association, behaviour-change strategies and technology-based strategies impact the escalation of physical activity awareness and compliance. They identify five key elements of behaviour-change strategies: (1) prompting a focus on previous success, (2) recognising and overcoming barriers, (3) using follow-up prompts, (4) providing guidance about where and when the action should be performed and (5) the timely analysis of behavioural objectives.^92^ In addition, using self-selected step goals or a regular steps target (e.g. 10,000 steps per day) were predictive of increased participation.^93^ Individuals are deemed ‘active’ if they complete a minimum of 10,000 steps per day, which equates to approximately 7.5 km.^94^ It, however, has recently been identified that a number greater than 7500 steps per day (5.7 km) appears to be adequate for individuals to achieve the recommended daily energy expenditure.^95^ Therefore, adults with type 2 diabetes should begin by setting achievable/feasible goals for steps per day before progressing to higher goals^96^ while aiming for at least 7500 steps per day.^97^

A recent systematic review and meta-analysis examined the effect of consumer wearable fitness trackers on physical activity and cardiometabolic health in people with a variety of chronic diseases.^98^ Individuals who used consumer wearable fitness trackers raised their physical activity level by 2123 steps per day, according to the analysis, which is comparable with 20 min or 1.5 km of walking.^98^ Similarly, it has suggested that wearing a pedometer is correlated with substantial rises in physical activity about 2000 steps or 1 mile of walking each day.^99^ Furthermore, the use of pedometers can be associated with clinically significant decreases in weight and blood pressure.^99^ The study discovered that setting a step goal and keeping a step diary could be important motivators for increasing physical activity.^99^ Recently, it has also been suggested that a physical activity intervention for individuals with type 2 diabetes, a gradual increase of the activity within the first 3 weeks, is expected to increase from moderate to vigorous physical activitycat the completion of therapy and follow-up.^100^ Technology-based strategies may be even more relevant considering the current pandemic. Internet-delivered physical activity promotion strategies could be more successful than conventional approaches for adults with type 2 diabetes, such as monitoring physical activity, goal setting and phone/e-mail support from a mentor; more research about social media approaches, however, is needed.^101,102^

## Conclusion

This review assessed the role of physical activity in people with type 2 diabetes in the context of the COVID-19 pandemic. Evidence suggests that exercise and physical activity have both physical and mental health benefits across a wide range of health conditions. For example, regular exercise can help reduce cardiovascular risk factors, such as diabetes mellitus and hypertension, and enhance patients’ health status and outcomes in people with established cardiovascular diseases. Even 10½ min of light-intensity physical activity has a beneficial impact in improving cardio metabolic health in individuals who cannot follow the guidelines recommendations for any reason. Likewise, achieving a daily step count of more than 7500 steps (5.7 km) appears to be sufficient for individuals to meet the recommended daily energy expenditure and lead to health benefits.

As a result of the current pandemic and the need for social distancing approaches, home-based exercise may influence anthropometric parameters associated with diabetes. This finding, while preliminary, suggests that home-based exercise could be an important element of future physical activity guidelines. Daily step counts and activity trackers can be beneficial in addressing several health and physical activity issues for the individual. It also presents a comprehensive context for tracking and prescribing physical activity. Together, the practice of physical exercise for treatment or prevention of type 2 diabetes is essential in all aspects of management, particularly during the COVID-19 pandemic. A gradual increase in exercise intensity is recommended for individuals beginning an exercise regimen, that is, more than 7500 steps until they are able to reach 210 min of moderate-intensity exercise or 125 min of vigorous-intensity exercise each week. The key element of prescribing physical activity as part of a patient’s management plan is understanding individual needs and aligning guidance with patient expectations and shared decision-making. Social and family support is also needed to maintain a commitment to medical treatment and physical activity to achieve optimal disease management and control.
